# Effect of a multimodal non-pharmacological intervention on older people with dementia: a single-case experimental design study

**DOI:** 10.1186/s12877-022-03501-w

**Published:** 2022-11-25

**Authors:** Kyosuke Yorozuya, Yoshihito Tsubouchi, Yuta Kubo, Yoshihiro Asaoka, Hiroyuki Hayashi, Takashi Fujita, Hideaki Hanaoka

**Affiliations:** 1 Faculty of Rehabilitation and Care, Seijoh University, 2-172 Fukinodai, 476-8588 Tokai, Aichi Japan; 2 Nara Gakuen University, Nara, Japan; 3 Department of Rehabilitation Technology, Toyokawa City Hospital, Toyokawa, Japan; 4 Graduate School of Biomedical and Health Science, Hiroshima University, Hiroshima, Japan

**Keywords:** Multimodal, Non-pharmacological interventions, Cognitive function, Nursing home, Dementia

## Abstract

**Background:**

Older people with dementia (PWD) in nursing homes (NHs) tend to have decreased cognitive function, which may cause behavioral and psychological symptoms of dementia (BPSDs) and hinder activities of daily living (ADLs). Therefore, taking measures against the cognitive decline of PWD in NH and, in turn, the decline of BPSDs and ADLs is crucial. The purpose of this study was to test whether a multimodal non-pharmacological intervention (MNPI) is effective in maintaining and improving global cognitive function, BPSDs, and ADLs in PWD in NHs.

**Methods:**

An intervention study using a single-case AB design was conducted in three subjects in NHs. During the non-intervention phase, participants underwent follow-up assessments, and during the intervention phase, they participated in an MNPI. The ABC Dementia Scale (which concurrently assesses ADLs [“A”], BPSDs [“B”], and cognitive function [“C”]) was used for the assessment.

**Results:**

One of the three patients showed improvement in dementia severity, global cognitive function, ADLs, and BPSDs. However, the other two participants showed no improvement following the MNPI, although the possibility of a maintenance effect remained.

**Conclusion:**

Although there is room for improvement of the MNPI, it may be effective in maintaining and improving cognitive function, ADLs, and BPSD, in PWD in NHs.

**Trial registration:**

The University Hospital Medical Information Network Clinical Trials Registry (http://www.umin.ac.jp/, No. UMIN000045858, registration date: November 1, 2021).

## Introduction

Cognitive function has been reported to be more prone to decline in older people with dementia (PWD) in nursing homes (NHs) than in older people living in the community [1–3]. Behavioral and psychological symptoms of dementia (BPSDs) have a variety of contributing factors (e.g., neurobiologically related disease factors, acute medical illness, unmet needs, pre-existing personality and psychiatric illness factors, caregiver factors, and environmental factors), and cognitive decline, a core symptom, has also been shown to be an important contributing factor [4–7]. The occurrence of BPSDs follows a vicious cycle in which cognitive decline accelerates, resulting in impaired activities of daily living (ADLs) and increased mortality [8–13]. In addition, even PWD who have the same cognitive dysfunctions may differ in the degree of specific cognitive dysfunctions, such as memory and orientation; accordingly, BPSDs and ADLs may also differ between individuals. Such BPSD and ADL disorders may lead to greater burdens on long-term care and medical and long-term care costs [14, 15]. Therefore, developing measures to maintain and improve the cognitive function of PWD in NHs is crucial.

Several interventions for cognitive decline and other dementia-related problems in PWD in NHs have been reported to be as effective as pharmacological interventions. However, pharmacological interventions do not provide adequate improvement of symptoms and are also associated with adverse effects, such as nausea and vomiting, diarrhea, weight loss, leg cramps, and increased mortality [16–20]. Therefore, non-pharmacological interventions (NPIs), which are considered as effective as pharmacological interventions, have become the first-line treatment option, although further development is required [2, 21].

In PWD, NPIs alone, such as reminiscence, music, and cognitive training, have been reported to improve cognitive function [22–24]. However, the effectiveness of a single NPI is limited; moreover, it is not effective in improving global cognitive function. Therefore, multimodal non-pharmacological interventions (MNPIs), which combine several NPIs to improve and maintain global cognitive function [25, 26], have recently received attention. A previous study reported that an MNPI that comprises exercise, cognitive training, and ADL training in PWD in NHs is effective in maintaining and improving cognitive functions, such as global cognitive function, executive function, attention, and memory [25]. However, few reports have focused on the effectiveness of MNPI in maintaining and improving the cognitive function of PWD in NHs, and the effectiveness of MNPI has not yet been fully verified. Therefore, we proposed a new MNPI based on previous research. Our newly designed MNPI consisted of a single 30-minute session that combined interventions related to exercise, cognitive function, and ADLs that had high clinical utility without the need for special equipment. Our aim was to determine the effectiveness of the proposed MNPI to enable its implementation as a valuable and clinically useful intervention that considers the burdens placed on PWD in NHs.

In this study, we aimed to test whether our MNPI, which we developed according to previous research, is effective in maintaining and improving global cognitive function in PWD in NHs, using a single-case experimental design (SCED). We also examined the effects of the MNPI on dementia severity, ADLs, and BPSDs.

## Methods

This study was conducted in accordance with the Single-Case Reporting Guideline in Behavioral Interventions (SCRIBE) 2016 Checklist [27].

### Design

Case series are suitable for assessing the feasibility of new interventions [28]. The MNPI in this study was a newly developed intervention based on previous research. Therefore, we decided to use a non-randomized single-case AB design, with phase A as the no-intervention phase and phase B as the intervention period, to evaluate the feasibility as well as the effect of the MNPI.

This study was registered in the University Hospital Medical Information Network Clinical Trials Registry (UMIN000045858; public title: A preliminary study of the effects of multimodal non-pharmacological interventions for PWD in NHs; registration date: November 1, 2021).

### Setting and study population

The participants were three PWD from two cooperating NH facilities. Inclusion criteria were individuals who (1) were aged 65 years or older; (2) had been admitted for at least 3 months; (3) had a Mini Mental State Examination-Japanese (MMSE-J) [29, 30] score of 23 or less with mild to moderate dementia according to the ABC Dementia Scale (ABC-DS) (which concurrently assesses ADLs [“A”], BPSDs [“B”], and cognitive function [“C”]) [31–38]; (4) were able to communicate and perform the tests and tasks; (5) provided consent or their family (or guardian) provided consent; and (6) had a diagnosis of Alzheimer’s disease or were suspected by a physician as having Alzheimer’s disease if the diagnosis was dementia. Exclusion criteria were individuals (1) with severe behavioral disorders or medical requirements; (2) with severe visual or hearing impairments; (3) who expressed their refusal to participate in the research; and (4) with cerebrovascular dementia, frontotemporal dementia, dementia with Lewy bodies, or other secondary dementias without a diagnosis of Alzheimer’s disease.

### Data collection

Basic characteristics of age, sex, marital status (married, widowed, divorced, or single), educational attainment (elementary school, junior high school, high school, or university), medication status, diagnosis of dementia, mode of transportation, and Clinical Dementia Rating (CDR) scores were collected for all participants from the medical records of the institutions. The Japanese version of the Neurobehavioral Cognitive Status Examination Five (COGNISTAT Five) was used to evaluate participants’ memory (short memory and recall), orientation, and construction ability. The COGNISTAT Five is a shortened version of the COGNISTAT [39, 40] that consists of four subsets: memory (word recall), score range 0–7; orientation, score range 0–12; construction, score range 0–6; and memory (delayed recall), score range 0–12 (higher scores indicate more severe impairment for word recall only). The validity and reliability of the Japanese version have been confirmed previously [41, 42].

### Multimodal non-pharmacological intervention

Previous research has suggested that MNPIs that combine exercise, cognitive tasks, and ADL training three times per week for 30 min per session for at least 8 weeks are recommended for PWD in NHs [25]. In addition, exercise and cognitive training are easy to introduce to PWD, even those with severe cognitive dysfunction; thus, structuring MNPIs around exercise and cognitive training may be useful in clinical practice [43]. According to the results of these previous studies, we proposed a specific and realistic MNPI based on the experiences of five occupational therapists (OTs) from a cooperating NH facility. In many of the previous studies, the duration of interventions ranged from 45 to 120 min per session, and the frequency was four or more times per week, which often presented difficulties in terms of time and labor to incorporate MNPIs into usual care practices. Therefore, we developed a highly effective MNPI that is short, simple, requires no special qualifications, and has the minimum time, frequency, and duration recommended in a previous systematic review [25].

The MNPI consisted of light-impact exercises, such as gymnastics and stretching, cognitive tasks, such as calculations and puzzles that could be performed in a short time, and ADL training, which included eating with chopsticks and spoons and changing clothes. In addition, tasks related to orientation, such as identifying the date, time, and place, were incorporated into the beginning and end of the session. Each intervention within the MNPI took approximately 10 min to perform, and the entire session was designed to be completed in 30–40 min (Table 1). The MNPI was usually administered as an individual intervention between 5:00 PM and 6:00 PM, which avoided the usual care time, and facility users were relatively free (the MNPI was administered individually to minimize group situations to prevent COVID-19 infection). The MNPI was implemented by the staff (OTs, physical therapists, and speech therapists) at each NH. The intervention period, phase B, lasted 8 weeks, and a total of 24 MNPI sessions were administered to each participant. The details of the MNPI were shared with the representative OTs at each site prior to the start of the study via materials and online meetings to ensure uniformity.

**Table 1:** Contents of the multimodal non-pharmacological intervention

Time frame	Component	Examples of the content of the intervention
10–15 min	Reality orientation and exercises	Reality orientation: confirmation of the date, place, and weather Exercises: gymnastics, stretching, and strength training
10 min	Cognitive activation	Calculations, card games, finger gymnastics, and puzzles
10–15 min	ADL training and reality orientation	Manipulation of jackets and trousers (simulated), use of chopsticks and spoons to transfer beans to a bowl, and brushing hair and teeth (simulated) Reality orientation: confirmation of the date, place, and weather, and reviewing the implemented program

ADL, activities of daily living

### Outcome measure

#### Assessments of dementia severity, global cognitive function, ADLs, and BPSDs

We used the ABC-DS to assess dementia severity. The ABC-DS is a dementia assessment scale that was developed in Japan that evaluates patients by asking their caregivers who have knowledge of the patient’s condition [31–38]. Questions included, “How well does the patient change their clothes?” and “How well can the patient remember the location of a familiar item?” Each of the 13 items is scored from 1 to 9 points. The maximum total score is 117 points and represents the severity of dementia. In addition, the maximum total score of the six items on ADLs is 54 points and represents the individual’s ability to carry out ADLs. The maximum total score for the three BPSD items is 27 points and represents the severity of BPSDs. The maximum total score for the four cognitive function questions is 36 points and represents global cognitive function. Higher total and individual item scores indicate milder dementia. The validity and reliability of the test have been confirmed previously. Furthermore, because the assessment is conducted by interviewing the caregiver, it places little burden on the patient. Another advantage of the ABC-DS is that the assessment takes only 10 min. Because the ABC-DS is not affected by learning effects, it was administered approximately once a week (18 times in total) from the time of participant recruitment to the end of the follow-up survey. The ABC-DS was administered by the staff (OTs, physical therapists, and speech therapists) at each NH.

#### Blinding

This study used an open-label, single-case design to prevent new coronavirus infections. Wherever possible, different people carried out the evaluation and interventions.

### Statistical analysis

There were 18 observation points of the ABC-DS (total score and scores for each item) time-series data for each participant, and therefore we applied the Bayesian unknown change-point (BUCP) model [44, 45] to analyze the effect of the MNPI on each participant. This analysis is applicable if there are at least three observation points in each phase, and more stable estimates are possible if there are more than eight observations in each phase [44]. The observed outcome variables (the total score and item scores of the ABC-DS) were assumed continuous and normally distributed. The analysis yielded the parameters *β*_11_, *β*_21_, *σ*, and *ρ*. *β*_11_ represents phase A, *β*_21_ represents phase B, *σ* represents the standard deviation (SD), and *ρ* represents the autocorrelation. For parameter estimation, Bayesian estimation and the Markov chain Monte Carlo method (MCMC) was used, and MCMC sampling was set to 120,000 times (chain = 4, burn-in = 5000). The change point (CP) detects where the change in the relationship between the explanatory variable and the objective variable occurs at a continuous time point. The effect size (*es)* in the two phases was calculated as the standardized mean difference of the intercept estimates

$$\it es=\frac{{\beta }_{11}-{\beta }_{21}}{{\sigma }_{\beta }}.$$

The 95% Bayesian confidence interval (CI) of the posterior distribution of the standardized mean difference determined the 95% limits of the credible value of the effect size under this distribution. Results were considered significant if the 95% Bayesian CIs of each estimated posterior distribution for phases A and B did not overlap, and the 95% Bayesian CI of the posterior distribution of the effect size was considered significant if it did not include zero [45].

For convergence determination, MCMC was considered to have converged to a steady state when the potential scale reduction factor (PSRF) was < 1.05 [46]. When the posterior distribution converged to a stationary state, the analysis was judged to have been performed appropriately with the assumed probability distribution (i.e., normal distribution).

The statistical software R (version 4.0.5; R Foundation for Statistical Computing, Vienna, Austria) with the runjags (version 2.2.0–2) and rjags (version 4–10) packages were used for all statistical analyses.

## Results

The estimation result using the BUCP model had a PSRF of < 1.05 for all posterior distributions, and MCMC was regarded as having converged to a steady state. Participants 1 and 2 completed all 24 MNPI sessions. Participant 3 completed 23 sessions because of the potential influence of the new coronavirus infection. None of the participants experienced any adverse events during the study period.

### Demographic characteristics

Participants were three PWD (participants 1–3: women, aged 92, 87, and 85 years, respectively) selected from two NHs who received the MNPI between November 2021 and May 2022. Participants 1 and 3 had a diagnosis of Alzheimer’s disease, and participant 2 had a diagnosis of dementia. All participants were taking anti-dementia medications. MMSE-J scores of the three patients were 21, 16, and 9, respectively, and the CDR scale score was 2 for all three participants. All patients had moderate dementia severity based on the initial ABC-DS (Table 2).

**Table 2:** Characteristics of the study participants

	Participant 1	Participant 2	Participant 3
Demographic characteristics			
Age, year	92	87	85
Sex	Female	Female	Female
Education	Unknown	High school	Junior high school
Diagnosis of dementia	Alzheimer’s disease	Dementia	Alzheimer’s disease
Length of stay, months	15	16	21
Marital status	Widowed	Widowed	Widowed
Antidementia drug	Yes	Yes	Yes
Locomotion	walking frame	walking frame	cane
Initial assessment			
MMSE-J	21	16	9
CDR	2	2	2
COGNISTAT Five			
Short term memory	3	7	2
Orientation	3	6	4
Construction	3	0	0
Recall	0	0	5
ABC-DS			
Dementia severity (total score)	78	71	81
ADLs	39	40	41
BPSDs	26	24	24
Cognitive function	13	7	16

MMSE-J, Mini Mental State Examination Japanese; CDR, Clinical Dementia Rating; COGNISTAT Five, The Japanese version of the Neurobehavioral Cognitive Status Examination Five; ABC-DS, ABC Dementia Scale; ADLs, activities of daily living; BPSDs, behavioral and psychological symptoms of dementia

### Results of the estimates for ABC-DS using the BUCP model

**Fig. 1:**
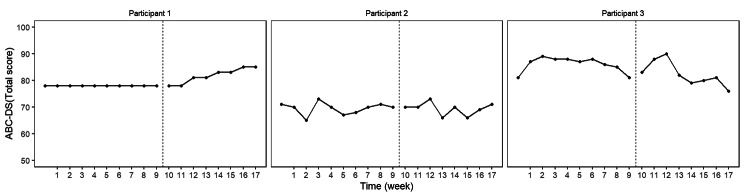
ABC Dementia Scale (ABC-DS) total score plot of each participant

**Fig. 2:**
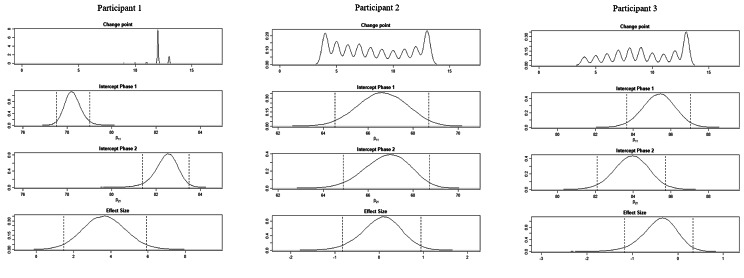
Plot of the estimates of the ABC Dementia Scale (ABC-DS) total score using the Bayesian unknown change-point (BUCP) model of each participant. The BUCP results of the participants are shown from the top in order of change point, phase A (intercept phase 1) posterior distribution, phase B posterior distribution (intercept phase 2), and effect size posterior distribution. The vertical dotted lines on either side of the posterior distributions are 95% Bayesian confidence intervals

**Fig. 3:**
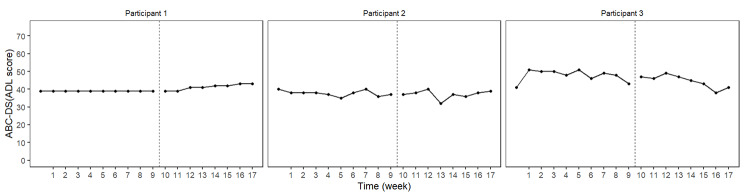
ABC Dementia Scale (ABC-DS) activities of daily living (ADL) score plot of each participant

**Fig. 4:**
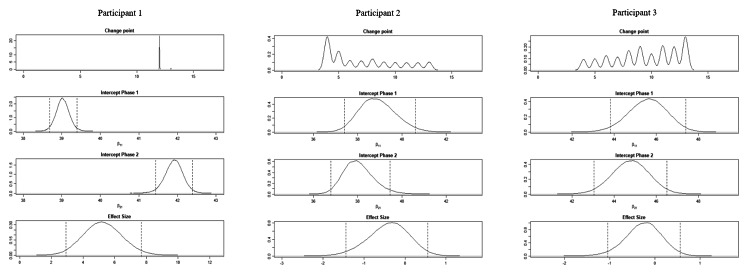
Plot of the estimates for the ABC Dementia Scale (ABC-DS) activities of daily living score using the Bayesian unknown change-point (BUCP) model of each participant. The BUCP results of the participants are shown from the top in order of change point, phase A (intercept phase 1) posterior distribution, phase B posterior distribution (intercept phase 2), and effect size posterior distribution. The vertical dotted lines on either side of the posterior distributions are 95% Bayesian confidence intervals

**Fig. 5:**
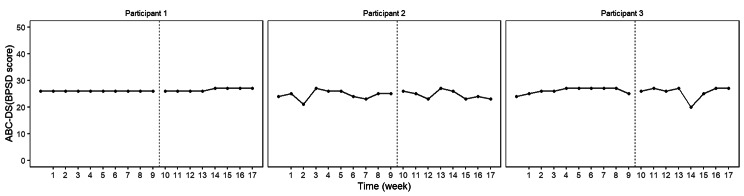
ABC Dementia Scale (ABC-DS) Bayesian unknown change-point (BUCP) score plot of each participant

**Fig. 6:**
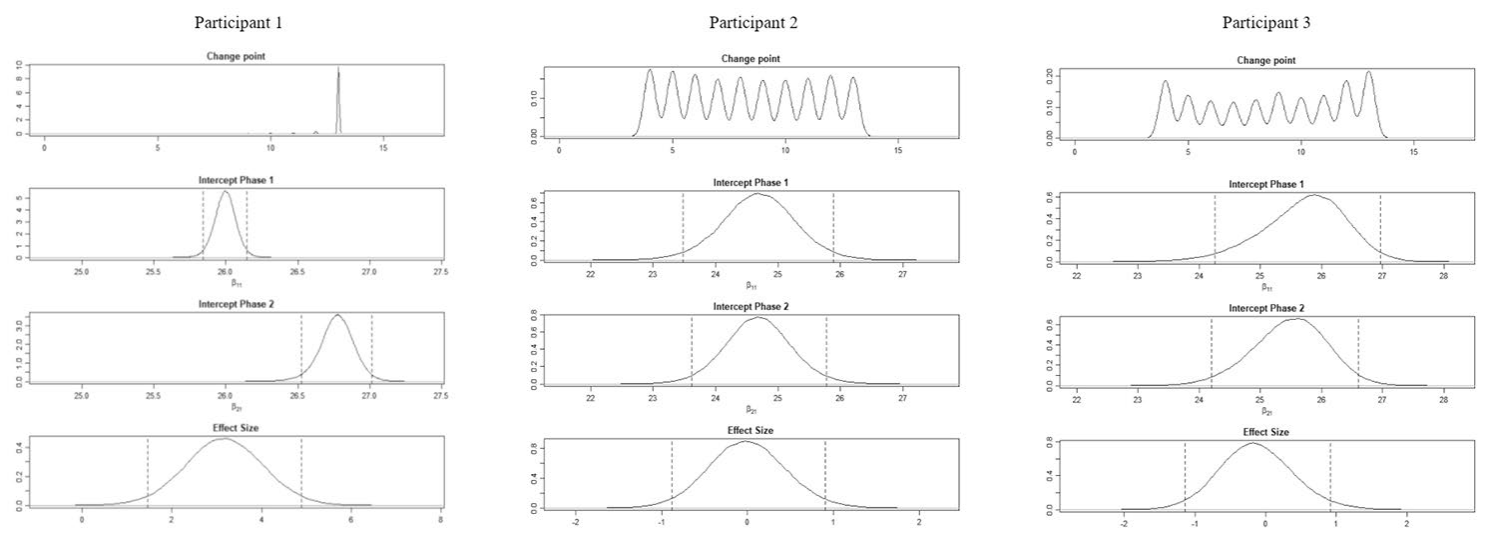
Plot of estimates of the ABC Dementia Scale (ABC-DS) Bayesian unknown change-point (BUCP) score using the BUCP model of each participant. The BUCP results of the participants are shown from the top in order of change point, phase A (intercept phase 1) posterior distribution, phase B posterior distribution (intercept phase 2), and effect size posterior distribution. The vertical dotted lines on either side of the posterior distributions are 95% Bayesian confidence intervals

**Fig. 7:**
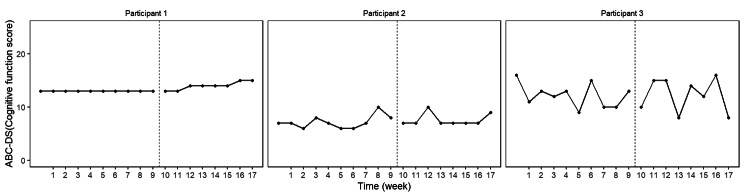
ABC Dementia Scale (ABC-DS) cognitive function score plot of each participant

**Fig. 8:**
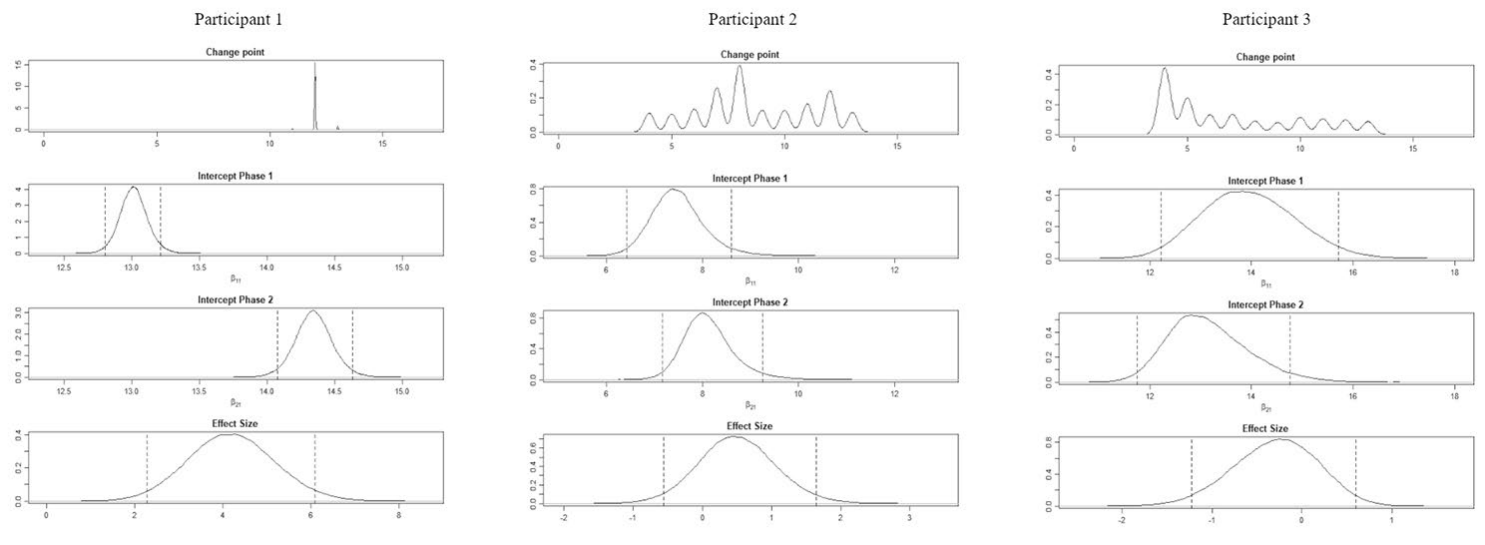
Plot of the estimates of the ABC Dementia Scale (ABC-DS) cognitive function score using the Bayesian unknown change-point (BUCP) model of each participant. The BUCP results of the participants are shown from the top in order of change point, phase A (intercept phase 1) posterior distribution, phase B posterior distribution (intercept phase 2), and effect size posterior distribution. The vertical dotted lines on either side of the posterior distributions are 95% Bayesian confidence intervals

The BUCP model results for the ABC-DS in participant 1 showed that the mean (standard deviation [SD]) CP was 12.14 (0.45) weeks for total score (severity), 12.03 (0.17) weeks for ADLs, 12.93 (0.43) weeks for BPSDs, and 12.04 (0.23) weeks for cognitive function. The estimated posterior distributions (95% Bayesian CIs) for the scores of phases A (*β*_11_) and B (*β*_21_) did not overlap for any of the ABC-DS items. For the 95% Bayesian CIs of the effect size, none of the items contained zero, and the effect of the intervention was considered statistically significant (Table 3; Figs. 1, 2, 3, 4, 5, 6, 7 and 8).

The BUCP model results for the ABC-DS in participant 2 showed that the mean (SD) CP was 8.36 (3.20) weeks for total score (severity), 7.16 (2.99) weeks for ADLs, 8.40 (2.93) weeks for BPSDs, and 8.62 (2.56) weeks for cognitive function. The estimated posterior distributions (95% Bayesian CIs) for the scores of phases A (*β*_11_) and B (*β*_21_) overlapped for all ABC-DS items, which indicated that there was no difference in the distribution of phases A and B in participant 2. The 95% Bayesian CIs of the effect size included zero for all items, and the effect of the intervention was considered non-significant (Table 3; Figs. 1, 2, 3, 4, 5, 6, 7 and 8).

The BUCP model results for the ABC-DS in participant 3 showed that the mean (SD) CP was 9.33 (2.87) weeks for total score (severity), 9.43 (2.79) weeks for ADLs, 8.75 (3.08) weeks for BPSDs, and 7.04 (2.99) weeks for cognitive function. The estimated posterior distributions (95% Bayesian CIs) for the scores of phases A (*β*_11_) and phase B (*β*_21_) overlapped for all ABC-DS items, which indicated that there was no difference in the distribution of phases A and B in participant 3. The 95% Bayesian CIs of the effect size included zero for all items, and the effect of the intervention was determined to be non-significant (Table 3; Figs. 1, 2, 3, 4, 5, 6, 7 and 8).

**Table 3:** Estimated result for each ABC-DS item score using the BUCP model

		Total score (severity)					ADLs					BPSDs					Cognitive function			
		Mean (SD)		95% Bayesian CI			Mean (SD)		95% Bayesian CI			Mean (SD)		95% Bayesian CI			Mean (SD)		95% Bayesian CI	
Participant 1	CP	12.14	(0.45)				12.03	(0.17)				12.93	(0.43)				12.04	(0.23)		
	${\beta }_{11}$	78.25	(0.38)	[77.52, 79.00]			39.03	(0.18)	[38.68, 39.39]			25.99	(0.08)	[25.84, 26.14]			13.01	(0.10)	[12.81, 13.21]	
	${\beta }_{21}$	82.46	(0.53)	[81.38, 83.49]			41.91	(0.24)	[41.44, 42.38]			26.77	(0.12)	[26.52, 27.02]			14.35	(0.14)	[14.08, 14.63]	
	$\sigma$	1.24	(0.31)	[0.75, 1.84]			0.57	(0.12)	[0.37, 0.55]			0.26	(0.06)	[0.16, 0.37]			0.33	(0.07)	[0.21, 0.47]	
	$\rho$	−0.001	(0.02)	[− 0.04, 0.03]			0.00	(0.02)	[− 0.03, 0.03]			0.00	(0.01)	[− 0.02, 0.02]			0.00	(0.03)	[− 0.05, 0.06]	
	*es*	3.65	(1.13)	[1.47, 5.91]			5.25	(1.22)	[2.93, 7.67]			3.17	(0.87)	[1.46, 4.89]			4.20	(0.97)	[2.28, 6.09]	
Participant 2	CP	8.36	(3.20)				7.16	(2.99)				8.40	(2.93)				8.62	(2.56)		
	${\beta }_{11}$	66.63	(1.08)	[64.52, 68.70]			38.91	(0.83)	[37.40, 40.61]			24.69	(0.60)	[23.48, 25.89]			7.48	(0.55)	[6.43, 8.61]	
	${\beta }_{21}$	66.84	(0.99)	[64.88, 68.73]			38.04	(0.69)	[36.80, 39.47]			24.68	(0.54)	[23.62, 25.77]			8.13	(0.54)	[7.16, 9.26]	
	$\sigma$	3.53	(0.68)	[2.36, 4.89]			2.35	(0.52)	[1.47, 3.41]			1.84	(0.36)	[1.21, 2.55]			1.39	(0.33)	[0.84, 2.04]	
	$\rho$	0.06	(0.05)	[− 0.05, 0.17]			−0.02	(0.06)	[− 0.14, 0.11]			0.01	(0.08)	[− 0.15, 0.16]			0.02	(0.18)	[− 0.33, 0.39]	
	*es*	0.06	(0.45)	[− 0.83, 0.95]			−0.40	(0.51)	[− 1.44, 0.56]			0.00	(0.45)	[− 0.88, 0.90]			0.51	(0.56)	[− 0.56, 1.64]	
Participant 3	CP	9.33	(2.87)				9.43	(2.79)				8.75	(3.08)				7.04	(2.99)		
	${\beta }_{11}$	85.37	(0.86)	[83.65, 87.04]			45.59	(0.92)	[43.82, 47.39]			25.71	(0.70)	[24.26, 26.97]			13.95	(0.91)	[12.23, 15.72]	
	${\beta }_{21}$	83.92	(0.93)	[82.09, 85.73]			44.78	(0.89)	[43.05, 46.51]			25.48	(0.62)	[24.20, 26.61]			13.15	(0.79)	[11.74, 14.76]	
	$\sigma$	3.85	(0.58)	[2.90, 4.99]			3.48	(0.59)	[2.43, 4.69]			1.96	(0.39)	[1.29, 2.76]			2.98	(0.62)	[1.89, 4.27]	
	$\rho$	0.04	(0.05)	[− 0.06, 0.14]			0.18	(0.09)	[0.01, 0.36]			−0.02	(0.09)	[− 0.19, 0.15]			−0.20	(0.17)	[− 0.53, 0.16]	
	*es*	−0.40	(0.38)	[− 1.17, 0.33]			−0.25	(0.41)	[− 1.05, 0.56]			−0.13	(0.53)	[− 1.14, 0.92]			−0.30	(0.47)	[− 1.23, 0.60]	

ADL, activities of daily living; BPSDs, behavioral and psychological symptoms of dementia: SD: standard deviation; CI, confidence interval; CP, change point; *es*, effect size

## Discussion

The purpose of this study was to evaluate the effect of our proposed MNPI on the cognitive function of PWD in NH using a SCED. In recent years, several studies using a SCED have proposed analyses using statistical methods in addition to visual inspection, attracting increased attention [45]. Because the ABC-DS is not impacted by learning effects, the frequency of assessment required for use of the BUCP model can be achieved even over a short period. Moreover, the Bayesian framework allows “acceptance” of the null or alternative hypothesis. Thus, unlike the classical framework, the possibility of no effect of this intervention can be deduced, even if the result is non-significant [45]. Therefore, the analysis using the BUCP model was suitable for this study.

### Effect of the MNPI on participant 1

The MNPI was effective in improving the ABC-DS scores (dementia severity, ADLs, BPSDs, and cognitive function) in participant 1. CP was detected approximately 2 weeks after the start of the intervention (12 weeks after the start of the study), which suggested that the effect emerged gradually from this period. Participant 1 had been in the NH for 15 months, and her condition was stable with no major changes in medication status, including during the study period. Therefore, the MNPI likely improved the score of each ABC-DS item. The results of participant 1 supported our hypothesis that the MNPI would be effective in improving global cognitive function, ADLs, and BPSDs in PWD in NHs.

### Effect of the MNPI on participant 2

The model BUCP model results for the ABC-DS in participant 2 showed that none of the items improved. Although the expected value of the CP was generally estimated at the intervention time point (i.e., 7–8 weeks after the start of the study), there were no obvious changes in any of the items, as shown in Figs. 1, 2, 3, 4, 5, 6, 7 and 8. The results of the MNPI for participant 2 indicated that the intervention did not improve dementia severity, ADLs, BPSDs, or cognitive function.

### Effect of the MNPI on participant 3

The results of the BUCP model for the ABC-DS in participant 3 showed that none of the items improved. Although the expected value of the CP was generally estimated at the intervention time point (i.e., 7–9 weeks after the start of the study), we detected no obvious changes in any of the items, as shown in Figs. 1, 2, 3, 4, 5, 6, 7 and 8. The results of the MNPI for participant 3 indicated that the intervention did not improve dementia severity, ADLs, BPSDs, or cognitive function.

### The effectiveness of the MNPI

In this study, all participants continued to engage in recreational activities and exercises as a daily routine, and no additional interventions or major care policy changes were introduced other than the MNPI. Therefore, the results were likely attributed to the MNPI. The effectiveness of the MNPI was confirmed in participant 1. However, the validity of our MNPI was not confirmed in participant 2 or 3. In contrast to participant 1, the ABC-DS phase A of participants 2 and 3 varied each week. It is also possible that factors that were not controlled for in this study, such as differences in the care policies of the target NHs at the time of the evaluation (participant 1 was from a different NH), may have affected the results. In addition, because participants 2 and 3 had a more unstable dementia status than participant 1, it may have been useful to consider tasks and BPSDs according to dementia status when administering the MNPI. Incorporating these into future MNPIs may enable greater efficacy. However, it is also possible that in PWD in NHs, cognitive function declines over a short period, such as that of the current study [1–3]. Therefore, the condition of participants 2 and 3 may have been maintained by the MNPI.

The results of our study suggest that our MNPI works to maintain or improve the severity of dementia, global cognitive function, ADLs, and BPSD in PWD in NHs. However, only one of three participants showed an improvement in their ABC-DS score, which suggested that the time, frequency, and duration of our MNPI design were inadequate. A review of the intervention period, time per session, and frequency of implementation should be considered. Consideration should also be given to the intervention provider adjusting the difficulty of the intervention according to the core symptoms, BPSD, or ADL impairments of the participant. The effectiveness of the MNPI for improving dementia severity, global cognitive function, ADLs, and BPSD in PWDs in NHs then requires examination in a larger number of participants. In the future, the problems of our MNPI identified in the present study should be addressed to enable a comparison between the AB and AA conditions (i.e., subjects participate in the non-intervention phase only) or a two-group design study to examine the maintenance and improvement effects of the MNPI.

The fact that the MNPI implemented in this study did not result in a worsening of ABC-DS score or other adverse events indicates that the MNPI can be safely implemented in NHs and is a useful intervention. Our MNPI was implemented using a minimal intervention design because our priority was to ensure clinical practicality in NHs. However, the time, frequency, and duration of the MNPI may need refinement to optimize the improvement of dementia symptoms. Nevertheless, we believe that our findings are valuable for clinical practitioners in NHs because our MNPI is a feasible intervention that can be implemented in NHs that has a small chance of slowing the progression of dementia symptoms.

### Limitations

Proxy-rated instruments, such as the ABC-DS, have lower reliability in measuring cognitive function than performance tests, such as the MMSE-J. Therefore, it is necessary to validate our findings using performance tests in the future while taking learning effects into consideration. Cognitive function in PWD in NHs may decline, even 8 weeks after admission [3]. Moreover, it is thought that the longer the length of stay, the more likely that decline will occur. In our AB design, the non-intervention phase was 9 weeks, and it is uncertain whether cognitive function would have declined if no intervention was provided after the intervention period of 10 weeks. Furthermore, we did not include a control group. Therefore, the maintenance effects and delays in the decline of cognitive function could not be determined. In addition, the possibility that the improvement effect in participant 1 was due to natural improvement cannot be ruled out completely. Nevertheless, our results provide a basis to further develop our MNPI and examine its effectiveness using a study design that offers a higher level of evidence.

## Conclusion

Our MNPI may be effective in maintaining and improving cognitive function, ADLs, and BPSD in PWD in NHs. However, refinement of the frequency, duration, and intervention period is required. Moreover, consideration of the intervention provider adjusting the difficulty of the intervention according to the observed impairment of the patient may be necessary.

## Data Availability

Because of privacy and ethical concerns, the datasets generated and analyzed during the current study cannot be made publicly available.

## List of abbreviations

PWD: older people with dementia.
NH: nursing home.
BPSD: behavioral and psychological symptoms of dementia.
ADL: activities of daily living.
NPI: non-pharmacological intervention.
MNPI: multimodal non-pharmacological intervention.
SCED: single-case experimental design.
SCRIBE: Single-Case Reporting Guideline in Behavioural Intervention.
MMSE-J: Mini Mental State Examination-Japanese.
ABC-DS: ABC Dementia Scale.
CDR: Clinical Dementia Rating.
COGNISTAT Five: Japanese version of the Neurobehavioral Cognitive Status Examination Five.
OT: occupational therapist.
BUCP: Bayesian unknown change point.
MCMC: Markov chain Monte Carlo method.
CP: change point.
CI: confidence interval.
PSRF: potential scale reduction factor.
SD: standard deviation.
